# Supplementary material for: A GNSS-based method to define athlete manoeuvrability in field-based team sports
Source: PLoS One. 2021 Nov 19;16(11):e0260363. doi: 10.1371/journal.pone.0260363 (PMC8604331; doi:10.1371/journal.pone.0260363)
Supplement: Attachment — Submitted filename: Responses to Reviewers.docx [file pone.0260363.s001.docx]

**Manuscript ID:** PONE-D-21-13310

**Title:** A method to define athlete manoeuvrability in team sports

**Comments to the editor**

The authors wish to thank the editorial team for the thorough review of this manuscript. All the comments provided were extremely helpful, and as such we believe the amendments made have increased the quality of this manuscript.

**Reviewer 1**

Comments to the Author

Reviewer #1: The study is interesting and provides important and new information around the studied subject. It is very well designed, and the methods are quite clearly presented. However, it is important to provide more details about the variables/metrics analyzed since they are not usual in sport science studies. The outcomes become clearer in the discussion section, which was well constructed, but the results section should better explore and detail the data analyzed. A final paragraph with a brief summary of the conclusive statements and possible implications is required at the end of the study.

Response: The authors wish to thank you for your time in reviewing this manuscript, it is greatly appreciated. All comments were helpful, and the suggested amendments have improved the quality of this manuscript. We have addressed the main points you have stated. Thank you again.

Abstract

The abstract was well developed and provides a good summary of the study. However, instead of explaining how each variable was calculated, it is important to describe “what” the variables represent. For example, the authors showed that “Compared to AFL, NRL had a greater negative quadratic coefficient…” but it is not clear what represents a “negative quadratic coefficient.” Is it related to good or bad manoeuvrability? This is essential to provide readers with a basic view of the rationale behind the study.

Response: Thank you for highlighting this with us. As we have tried to summarise a novel concept, we have had to include more detail regarding the methods as possible. However, we agree that insufficient information was included about what our findings mean. Some information about the calculation has been removed, and additional text has been included about what tortuosity represents, as well as in the results what a negative quadratic coefficient means. We hope these changes meet your expectations.

It is also important to briefly describe how many matches of each sport were analyzed.

Response: Thank you for highlighting this to us. This information has now been included; “62 athletes (Australian Football League [AFL]; n = 36, 17 matches, National Rugby League [NRL]; n= 26, 21 matches).”

Introduction

Lines 18-19? It is important to provide a reference to support this statement.

Response: The authors have added the word ‘anecdotally’ here, as what we are discussing is more of a conceptual background of the movements that we are attempting to quantify. Further, we can’t find any research which specifically prove this statement, however our statement is logical in this context.

**Response:**

Lines 19-21: The “gamespeed” concept of Prof. Ian Jeffreys could be helpful to better support this idea (please, see some references below).

Jeffreys, I., Huggins, S., & Davies, N. (2018). Delivering a gamespeed-focused speed and agility development program in an English Premier League Soccer Academy. Strength & Conditioning Journal, 40(3), 23-32.

Jeffreys, I. (2010). Gamespeed: Movement training for superior sports performance. Coaches Choice.

Response: Thank you for this suggestion. Based on the contents of each article, we have included the first one as a reference for our statement regarding movements in the game as we thought this was most relevant here.

Lines 22-24: This comparison is interesting, but maybe it would be better to use a reference more related to the sport science context. The “gamespeed” concept can also be used in this regard.

Response: We appreciate this suggestion, however as we are linking to the calculation of tortuosity and its application in other contexts (outside of sport) we believe it is most appropriate as It currently stands. As tortuosity has been researched in other context, we have emphasized this more, therefore have included an additional sentence; “Manoeuvrability has been extensively researched in other contexts including animal behaviour (13), aerodynamics (14) and vehicle design (15) but is also applicable in a sporting context.” We hope this will suffice.

The introduction section was well designed, but before describing the purpose and objectives of the study, it is important to better emphasize the importance of assessing manoeuvrability in team sports and the practical relevance of this study to increase the body of knowledge on this topic.

Response: Whilst we agree with your statement here, throughout the introduction we have stated the practical relevance of manoeuvrability in team sports in small parts, therefore it may not have stood out. However, based on the suggestions of yourself and reviewer 2, we have attempted to increase this by including additional sentences throughout. We hope this has improved the introduction. Two of these sentences read;

“Perhaps, the ability to accurately quantify such movements may result in practitioners implementing these into training programs more frequently and specific to the demands of competition.”

“Potentially if this physical attribute was appropriately trained, this may be improvable and performance advantages may result.”

Methods

It is important to better characterize the subjects/teams analyzed, including more information regarding them. Were they from the first division? How many games did they participate during the analyzed season?

Response: Whilst we thank you for this suggestion. Based on your suggestion as well as reviewer 2, we have added considerable detail here. Specifically, the competitions (AFL/NRL) are the names of the respective competitions (i.e., the division) of the respective sports, therefore the terminology of the division isn’t applicable here. Regarding the number of games, this information is detailed in the ‘Global Navigation Satellite Systems and Data Analysis’ section, as we deem this the most appropriate spot. We have also provided additional information relating to the athletes’ positions and their training programs. We hope this clears up your concern.

Line 105: Please, delete “has been”.

Response: Thank you, this is deleted now.

Results

It is not completely clear what represents the “quadratic coefficient (a), the linear coefficient (b), or the intercept (c)” in relation to the manoeuvrability capacity. These analyses are not widespread and easy to understand (especially in sport science). Therefore, authors should better explain how these results are related to the study purposes. This is also essential to improve the impact and usefulness of the study for researchers and practitioners.

Response: We thank you for your comment here and we do understand your point. However, as results sections are simply for stating results and the discussion is intended to explain such results, we don’t believe here is appropriate to add any explanation. To help explain the findings, we have included a short explanation of what the quadratic coefficients mean, which has assisted in some explanation in the discussion section. This reads as “Specifically, a represents the overall position of the curve up and down the y axis (i.e., wide or narrow), b reflects the upward or downward linear trend in y values along the x axis, and c is a constant (intercept), representing where the relationship sits on the y axis.” We have discussed the applied nature of these findings in greater detail in the Discussion, where typical convention would dictate.

In the abstract section authors state that “A curvilinear relationship exists between maximal tortuosity and running speed, reflecting that as speed increases, athletes’ ability to deviate from a linear path is compromised.” However, this information is not clearly presented in the results section. It is important to provide more practical information for readers given the complexity of your analyses.

Response: Thank you for your comment here. We have included this information and further explanation of the findings in the discussion rather than the results. As per the journal guidelines, we have selected to have ‘separate’ results/discussion/conclusions, therefore any results explanation is situated within the discussion. As we understand your overall point, we have made substantial changes to the discussion to better link the findings to a practical interpretation. We hope these changes suffice.

Figure 3: Does this figure show data from a representative athlete or mean values? Please, clarify.

Response: Thank you for highlighting this to us. This data is the mean by sport and duration - this is now included in the figure caption.

Figure 4: It is not clear why authors presented this figure. It is not related to the study purposes. Please consider removing it.

Response: Although this figure is not depicting an actual result as such of our study, this figure helps present the practical application of the use of tortuosity. As we are presenting a new concept (manoeuvrability) in this study, we have attempted to provide as many practical applications as possible, therefore we feel that including an example of a rehab session versus a game is important. We hope you understand our reasoning here.

Since the outcomes presented here are not usual in sport science studies, it is important to provide some information about the reliability of the variables presented.

Response: Thank you for your comment here. The authors don’t fully understand what you’re referring to when you say ‘outcomes’. However, we assume you may be referring to the “validity” (i.e., accuracy) of GPS for monitoring players speed and position. As such, we have mentioned the validity of GPS for establishing XY position (line 311) and have also included a reference for the validity of GPS for monitoring speed. Please let us know if this is not what was being referred to.

Discussion

Line 182: It is not simple and practical and requires great experience in data analysis.

Response: The authors partially disagree with this statement, however, we have removed the word ‘simple’ from this sentence. The process involved is relatively straight forward for those involved or have experience using GNSS in applied settings. We agree that a degree of data analysis expertise is required, however this is a given within this applied cohort of practitioners.

Lines 183-185: “This has implications for training prescription and rehabilitation and although not examined here, potentially performance evaluation in some sports.” Based on the results, it is not possible to affirm this. Please consider reformulating this statement.

Response: Thank you for this comment. Whilst we take this comment on board, respectively we disagree, in that we are simply suggesting how tortuosity can be used beyond this paper in applied settings. As applied practitioners ourselves, this concept is one which we have already begun to implement within our programs, and tortuosity has provided us with an objective measure to quantify such movements.

Lines 188-190: “It was demonstrated that team sport athletes typically complete non-linear movements peaking at a speed of 2 m∙s^-1^, decreasing thereafter to approximately 5 m∙s^-1^ where tortuosity plateaus, and a linear running path is adopted.” This information should be presented in the results section. It is not clear how authors obtained this outcome.

Response: Whilst we understand your point here, we have provided adequate information surrounding the curve-linear relationship between speed and tortuosity and have presented our findings of this in multiple ways (Table 1, Figure 2, 3 and 4). As we have presented a new concept here, we tried not to fixate over the statistical findings (i.e., differences between sports), rather by presenting and explaining the data in a practical way. As such, we have taken this comment on board and have added additional text within the discussion on the interpretation of the relationship coefficients in table 1. We believe these additions have strengthened this section, so we thank you again for this contribution.

Line 197: Again, this is not simple and practical. Please consider reformulating this.

Response: We understand your perspective here, however as stated previously those working with GNSS data in professional settings require a standard of data analysis skills. To reflect this, we have included the wording “for those with appropriate expertise” into this sentence. We hope this addition is adequate.

Lines 206-208: This sentence is difficult to follow. Please, consider rephrasing this.

Response: Thank you for highlighting this issue with us. We agree it was worded poorly and has since been reworded to be clearer. Thank you.

Lines 260-262: Please provide a reference for this statement.

Response: Thank you, in support of the statement we have added the following reference:

Hickey, J. T., Timmins, R. G., Maniar, N., Williams, M. D., & Opar, D. A. (2017). Criteria for progressing rehabilitation and determining return-to-play clearance following hamstring strain injury: a systematic review. Sports medicine, 47(7), 1375-1387.

Line 265: I understand the importance of the discussion around the rehab process, but it is beyond the study purposes and the match analysis performed. As suggested for figure 4, consider removing this part.

Response: Thank you for the comment. While we agree quantifying the rehabilitation process was not a purpose of the study, we feel that the example of using tortuosity in relation to speed provides a practical context of quantifying manoeuvrability in team sports when developing a return to play program. We wanted to highlight the ability of GNSS devices to quantify more than just speed, high speed running, and acceleration. As such, the authors have added some of this to the conclusion requested in the next comment.

A conclusion is required to summarize the main outcomes found and reinforce the practical relevance of the study.

Response: Yes, we agree that there should be a concluding statement here. As such, between lines 338 and 345 is a concluding paragraph. Thank you for this suggestion.

Reviewer #2: Review manuscript number: PONE-D-21-13310

Title: A method to define athlete maneuverability in team sports

Comments and Suggestions for Authors

General comments

The article “A method to define athlete maneuverability in team sports” aimed to presented a method of quantifying the maneuverability of team sport athletes and investigated its relationship with running velocity during competition in team sports. I acknowledge the authors on their commitment to conduction such a difficulty study since working with athletes it is always hard. Also, I am pretty sure this topic is interesting and, indeed, more studies are required. However, I have major concerns regarding your study design and manuscript (listed below) which I think you should address thoroughly. I would be happy to review an updated version of your manuscript. following my comments and suggestions.

Response: The authors wish to thank you for your time in reviewing this manuscript, it is greatly appreciated. All comments were helpful, and the suggested amendments have improved the quality of this manuscript. We have addressed the main points you have stated and hope that this meets your requirements. Thank you again.

1) In general, with respect to the authors (effort and choices) the manuscript lacks methodological depth. Moreover, while reading your manuscript I feel a little lost (there is a lot of unhelpful information) ... Sometimes you were talking about differences in tracking systems…After you spoke about team sports physical demands… Change of direction/ reactive. What is your main objective???It is hard to follow…. Lot of information in the introduction

Response: Thank you for this comment. Based on this suggestion and that of reviewer 1, we have added some more content to the introduction to add clarity, in particular brief summarising sentences at the end of each paragraph. Each paragraph has a specific focus, and a purpose in relation to the new concept we are introducing in this manuscript. Regarding the differences in tracking systems, the introduction focuses solely on GNSS devices and no other tracking systems such as smart cameras and Local Position Systems, so we can’t provide any explanation for this point. Although there is a lot of information in the introduction, we feel that this is necessary as we are introducing a novel concept to team sports. In summary, our introduction includes a summary on; GNSS devices, movements within team sports, measuring these movements with GNSS,

2) I really want to know the main purpose of using Global navigation satellite system (GNSS)? is your study the first to use this kind of material? If yes (please give information about validity and reliability...); If no (What is the novelty of your study….)

Response: GNSS devices are used extensively within team sports, both in a professional and research setting. As discussed in the manuscript, the validity of GNSS devices has been previously quantified. The novelty of the study is the quantification of manoeuvrability, which has not been done before in a team sport setting

3) I noticed you used the term” team sports” in the title but you used only (football and rugby)?? No other disciplines?? … I think that the title is very general and do not reflect your methodology.

Response: Thank you, yes we agree that team sports is quite general and as such have changed the title to “A method to define athlete manoeuvrability in field based team sports”? Further to this, we have changed parts of the discussion which refer to team sports and have changed this to ‘two field based team sports’.

4) Physical and physiological demands of team sports vary from one discipline to another and it is very hard and not logical to give a general conclusion only by relying on two disciplines… Perhaps you could orient your study objective to compare only the requirements of Football and Rugby… it will be more relevant in my opinion.

Response: Thank you, and yes we agree that the demands of team sport vary, in particular between field and court based sports. Subsequently, and aligning with the title modification you suggested in your comment above, we have provided clarity throughout the paper in specifying that we were assessing two field based sports. We believe this has increased the quality of the manuscript.

5) You did not give additional information about the state of health of the participants during the whole sports season (injuries, illnesses ... which can stop sports practice) and of course it will influence your results.

Response: We have clarified that the players were monitored over the standard competition phase which consists of weekly games. As we only monitored competition games, players that were deemed fit to play were only included in the analysis. We hope this clarifies your concern.

6) You did not give additional information about the sports calendar of the participants during the whole sports season (congested game phase…Playoff... Covid19 lockdown) each phase has its own physical requirements and of course it will influence your results.

Response: While we agree that changes to the competition schedule may modify the physical requirements of players, during this study we were lucky enough in Australia to continue with regular weekly competition games in the AFL and NRL. Subsequently, we have highlighted that games were on a weekly basis for the collection of data for this research project. Additional text relating to the match recovery periods (turn around length) has also been added, reading; ”Matches were played weekly during the competition, with match recovery periods ranging from 4 to 10 days in AFL and 5 to 9 days for NRL.”

Specific comments

Introduction

1)The authors needed to provide a much more robust justification for this study based on the importance of (using such materiel); (why only) and finally what is new in your work (differences with previous studies investigating).

Response: Thank you for this comment. Throughout the introduction, the authors believe there is sufficient detail relating to the importance of quantifying manoeuvrability. We have attempted to highlight the gap in research as much as possible. Specifically, we believe lines 25-27, 37-39, 51, 57-58 and lines 63-66 now include such information you have suggested.

2) I suggest to add a clear hypothesis at the end based on what was previously reported (in the literature).

Response: Thank you for this suggestion. This has been included now.

Methods

1) Did you proceed with a priori power analysis?

Response: In this context, a priori power analysis is not relevant as we are limited by the number of athletes within each squad, a factor in which we can’t control. Further, as we aren’t using inferential statistics to explain differences between the quadratic equation coefficients, this priori analysis again is not applicable. As our total dataset is comprised of 714 match files (372 for AFL and 342 for NRL), we are confident that our sample size is adequate. We hope you understand our reasoning here.

2) Please add BMI and BF%

Response: In the context of both AFL and NRL, both BMI and BF% aren’t relevant anthropometric measures, and therefore not included in this study. Given the high muscle mass of these athletes compared to the general population, their BMI is typically categorised as ‘obese’ which is simply incorrect, and a limitation of this measure regarding extreme spectrums of muscle mass. Further, BF% is also non-applicable here as this cannot be measured reliability across the two sports, using the same tool (i.e., DEXA). Further, given the constraints applied to these athletes during the 2020 season and the restricted living and training environment, athletes weren’t able to have DXA scans or have skinfolds taken as this required additional staff or environments which weren’t permitted. As such, body mass and height are the most reliable metrics we have available which are included in this study.

3) Please add players training program/ History…

Response: Thank you for this suggestion. We have included a section regarding their training programs, reading; “Prior to and during the competitive season, athletes from both teams participated in a full-time professional training program. This entailed up to 4 field-based training sessions per week, undergoing specific skills-based training, as well as speed and conditioning training. Additionally, up to 4 resistance based sessions were completed, with a primary focus on strength and power development.” Nevertheless, it is important to note that we are showing this method as an exemplar, and the actual position differences and sport differences are somewhat secondary (specifically because we haven’t looked at the performance side here or tried to predict tortuosity from a feature set (i.e., using BMI, training history, etc). We hope this explanation addresses the concerns of the reviewer.

4)Please add the players position (it is important!!)

Response: Thank you raising this oversight with us. Positions have bene included, reading; “For both teams, athletes from all positional groups were included (despite no positional analyses conducted). The AFL squad included; midfielders (n=11), mobile backs (n=4), mobile forwards (n=9), ruck (n=1), tall back (n=4) and tall forwards (n=3). For NRL, the squad comprised of edge forwards (n=4), fullback (n=1), halves and hookers (n=6), middle forwards (n=9) and outside backs (n=6).”

5)Please add inclusion criteria…

Response: As we have conducted this within a professional setting, there was no set inclusion criteria’ as this is simply if they completed game. However, we have included some text to clarify this, which reads “Athletes were included in the study if they played a game for their respective team, and completed the match (i.e., were uninjured).”

Results

-The results of the manuscript are well presented (Nothing to address)

Response: Thank you kindly for the positive feedback on this section of the manuscript

Discussion

1)By following my suggestions (concerning adding a clear hypothesis) the authors may support or reject this hypothesis in the first part of the discussion…

Response: Thank you. We have since included the statement; “Our hypothesis was partially supported whereby there was a decrease in tortuosity as running speed increased.”

2) The authors provide the most relevant information about the topic in this part (good work). However, I advise the authors to avoid using long sentences… I suggest to reduce the length of this topic (please try to be selective) …

Response: Thank you for this suggestion. We have made this amendment throughout the discussion.

Conclusion

1) The most important question here is what is the novelty that your study gives to the field??

Response: The authors believe we have added sufficient information relating to the novelty of this study, not only in the discussion but throughout the entire manuscript. Specifically, in the introduction, lines 25-27, 37-39, 210-212, 221 to 223, 296-297,303 -306, and throughout the concluding paragraph. Overall, we believe these sections (some of which are new to this revision) provide this information. Thank you again.

2) Please add a practical applications part in which you may clearly explain how does the present study affect the field.”

Response: Thank you for this comment. Reviewer 1 had the same suggestion, and as such between lines 320 and 327 is a concluding paragraph.
