# Supplementary material for: A GNSS-based method to define athlete manoeuvrability in field-based team sports
Source: PLoS One. 2021 Nov 19;16(11):e0260363. doi: 10.1371/journal.pone.0260363 (PMC8604331; doi:10.1371/journal.pone.0260363)
Supplement: Attachment — Submitted filename: Response to Reviewers.docx [file pone.0260363.s002.docx]

Response to Reviewers Comments

Lines 57-58: This sentence is not clear. What would be improvable? The running speed or the control? Please restructure the sentence for the sake of clarity.

Response: We have now restructured this sentence to read ‘Potentially if manoeuvrability was appropriately targeted in training, it may be improvable and performance advantages may result’

The authors stated that they followed the journal guidelines to organize/divide the manuscript into results, discussion, and conclusions. Under this rationale, following the journal guidelines, the results section “should describe the results of the experiments”. Since figure 4 is not a “result” of the experiment, it should be removed from the study.

Response: We understand your point as Figure 4 is not a direct ‘result’ from a specific hypothesis/experiment, however it is a crucial element in demonstrating the practical application of the findings. As PLOS One is an applied journal that is highly relevant to practitioners working with athletes, we have decided to present figure 4 in the discussion (line 301) to provide an exemplar of the method and provide direction for future research.

Accordingly, as figure 4 is not part of the results of the study, the discussion regarding this respective figure should also be deleted.

Response: As mentioned above, we have moved figure 4 to the discussion and still believe that the associated discussion is relevant for the applied nature of PlosOne.

Reviewer #2: Review manuscript number: PONE-D-21-13310

The title is very general in my opinion and do not show the originality of your work... I suggest to use this "Using GNSS as a valid tool to quantify maneuverability in australian football and rugby leagues"

Response: We have considered the title and added the term ‘GNSS-based’ as we believe that this distinction further assists with the description of the paper. We do however see the potential for the method to be used in any running-based, outdoor team sports so would prefer that we did not delimit to the two exemplar sports on this occasion, so as to not limit its appeal, potential application and ability for citation elsewhere.

I suggest directing your objective to compare football and rugby in terms of maneuverability recorded by (GNSS) and the relation with physical requirements of each sporting discipline (maybe reported with time motions analysis)...

Response: We respectfully disagree as we believe that this is a secondary objective to the broader, more generalisable current aim. We use these two sports here to highlight the applicability of the method in two team sports, however the primary emphasis is on promotion of the method itself. Considering there is not a commonly reported (or from our practical experience, utilised) method similar to tortuosity we would maintain that it is justified that scope be broadly maintained as in the manuscript’s current format.

Suggest to discuss differences between football and rugby (maneuverability GNSS) and compare your results with other using different method...

Response: We believe that we have already directly compared the two sports on lines 245-259 and again at lines 286-293. We are not entirely clear on what the reviewer is requesting in the second part of this comment, however if they would be willing to clarify then we will be happy to address.
